# Comprehensive investigation of cytokine- and immune-related gene variants in HBV-associated hepatocellular carcinoma patients

**DOI:** 10.1042/BSR20171263

**Published:** 2017-12-12

**Authors:** Fengxue Yu, Xiaolin Zhang, Suzhai Tian, Lianxia Geng, Weili Xu, Ning Ma, Mingbang Wang, Yuan Jia, Xuechen Liu, Junji Ma, Yuan Quan, Chaojun Zhang, Lina Guo, Wenting An, Dianwu Liu

**Affiliations:** 1 Department of Science and Technology, The Hebei Key Laboratory of Gastroenterology, The Second Hospital of Hebei Medical University, Shijiazhuang, China; 2 Division of Epidemiology, School of Public Health, Hebei Medical University, Shijiazhuang, China; 3 Department of Pediatric Surgery, The Second Hospital of Hebei Medical University, Shijiazhuang, China; 4 Department of Central Laboratory, Shenzhen Following Precision Medical Research Institute, Shenzhen, Guangdong, China; 5 Department of Infectious Disease Control, The Second Hospital of Hebei Medical University, Shijiazhuang, China; 6 Department of Gastroenterology, The Second Hospital of Hebei Medical University, Shijiazhuang, China; 7 Department of Infectious Disease, Hebei Chest Hospital, Shijiazhuang, China; 8 Department of Central Laboratory, The Second Hospital of Hebei Medical University, Shijiazhuang, China

**Keywords:** cytokine, gene level association, HBV-HCC, immune genes, Target Region Sequencing (TRS)

## Abstract

Host genotype may be closely related to the different outcomes of Hepatitis B virus (HBV) infection. To identify the association of variants and HBV infection, we comprehensively investigated the cytokine- and immune-related gene mutations in patients with HBV associated hepatocellular carcinoma (HBV-HCC). Fifty-three HBV-HCC patients, 53 self-healing cases (SH) with HBV infection history and 53 healthy controls (HCs) were recruited, the whole exon region of 404 genes were sequenced at >900× depth. Comprehensive variants and gene levels were compared between HCC and HC, and HCC and SH. Thirty-nine variants (adjusted *P*<0.0001, Fisher’s exact test) and 11 genes (adjusted *P*<0.0001, optimal unified approach for rare variant association test (*SKAT-O*) gene level test) were strongly associated with HBV-HCC. Thirty-four variants were from eight human leukocyte antigen (HLA) genes that were previously reported to be associated with HBV-HCC. The novelties of our study are: five variants (rs579876, rs579877, rs368692979, NM_145007:c.*131_*130delTG, NM_139165:exon5:c.623-2->TT) from three genes (*REAT1E*, NOD-like receptor (NLR) protein 11 (*NLRP11*), hydroxy-carboxylic acid receptor 2 (*HCAR2*)) were found strongly associated with HBV-HCC. We found 39 different variants in 11 genes that were significantly related to HBV-HCC. Five of them were new findings. Our data implied that chronic hepatitis B patients who carry these variants are at a high risk of developing HCC.

## Introduction

Hepatitis B virus (HBV) affected more than 248 million individuals worldwide [1] and caused deaths of 500000–1.2 million each year [2]. The HBV infection outcomes including self-healing and persistent infection, are associated with viral factors, environmental factors, and host genetic factors [3]. Host immune responses, such as virus recognition, antigen processing and presentation, as well as immune regulation are associated with pathogen infection and clearance [4]. The innate immunity plays important roles in immunopathology and treatment of HBV infection [5]. Toll-like receptors (TLRs) and innate immune sensors of pathogen serve as immune regulators of both innate and adaptive immune responses [6]. Recent study showed that young mice with TLR4 mutation exhibit rapid HBV clearance [7]. NOD-like receptor (NLR), another immune sensor can sense and react to viral infection through the inflammasomes [8,9]. The adaptive immunity genes such as human leukocyte antigen (HLA), have been widely studied in the HBV infection by genome-wide association study (GWAS) [10–14]. Polymorphisms of some cytokine genes are also associated with the outcomes of patients with HBV infection [15–18]. With the advances in high-throughput sequencing (HTS), some rare variants have been found and associated with HBV infection. To identify the variants associated with outcomes of patients with HBV infection, we systematically sorted and sequenced 404 genes related to innate and adaptive immunity, cytokine and cytokine receptors, and immune regulator genes in Chinese population infected with/without HBV infection. The identification of variants associated with the outcome of patients with HBV infection helps physicians treat the patients more specifically.

## Materials and methods

### Study subjects and sample collections

Three groups were included in the present study: hepatocellular carcinoma (HCC) group individuals who had HBV infection history and self-healed (self-healing (SH)) and healthy controls (HCs). The participants were from the First, Second, Third, and Fourth Hospital of Hebei Medical University (Shijiazhuang, China) and the Fifth Hospital of Shijiazhuang (Shijiazhuang, China) between January 2015 and January 2016. HCC diagnosis was according to the guidelines of the America Association for the Study of Liver Diseases (AASLD) [19]. HCC patients were seropositive for HBsAg or had HBV infection history. Self-healing HBV patients had no previous HBV immunization and the liver enzymes were normal, seropositive for HBsAb and HBeAb, and seronegative for HBsAg, HBeAg, and HBV DNA. HCs had normal liver enzymes, seronegative for HBcAb, HBeAb, HBeAg, HBsAb, and HBsAg, no previous HBV immunization, and no endocrine, cardiovascular, and kidney diseases, and other liver diseases history. All participants had written informed consents. The protocol of the present study was in accordance with the Declaration of Helsinki, and was approved by the Human Ethic Committee of the Second Hospital of Hebei Medical University. Five milliliters of whole blood were drawn and stored at −80°C until use. DNA was extracted from whole blood samples using an Invitrogen PureLink Genomic DNA Mini Kit (Thermo Fisher, Foster City, CA, U.S.A.).

### Cytokines and immune genes search and gene panel design

The cytokines and cytokine receptors, TLRs, NLRs, HLA family genes, T-cell activation, and co-stimulation genes, natural killer cell target genes, G-protein-coupled receptor genes such as free fatty acid receptor (FFAR), hydroxy-carboxylic acid receptor (HCAR) family genes were sorted according to the HUGO Gene Nomenclature Committee (HGNC) database [20]. TargetSeq™ (iGeneTech, Beijing, China), an RNA probe based liquid phase chips were used to capture the whole exome region of selected genes.

### Targetted region sequencing

The concentration of DNA was determined using a Qubit dsDNA BR Assay Kit (Thermo Fisher, Foster City, CA, U.S.A.). Two microliters of sample DNA were used for sequencing. Using an automated SPRIworks System (Beckman Coulter, San Jose, CA, U.S.A.), the 200–500 bp size libraries were constructed with a TruSeq DNA Sample Preparation Kit (Illumina, San Diego, CA, U.S.A.). Then the small (200–500 bp) size libraries were further used for capturing using TargetSeq™ liquid chip capture sequencing kits (iGeneTech, Beijing, China) according to the manufacturer’s instructions. In short, before the hybrid capture, small size libraries were mixed with Hybridization Block (iGeneTech, Beijing, China) to avoid any repeating sequences to form hybrid itself. And then, melting the Hybridization Buffer at room temperature and preheating Hybridization Buffer in 65°C water bath. After the solution completely dissolved, 20 µl Hybridization Buffer was mixed with 20 μl small-size library in a PCR tube, and 5 µl RNase block (Thermo Fisher, Foster City, CA, U.S.A.) was mixed with ssRNA probe to prevent probe degradation. The purpose of liquid phase hybrid was to capture objective DNA fragments by ssRNA probe complementary pairing. The Hybridization capture was performed in ABI 2720 PCR (Thermo Fisher, Foster City, CA, U.S.A.) at 65°C overnight for incubation. After hybridization, the DNA:RNA hybrid was enriched by biotin-labeled magnetic beads (Thermo Fisher, Foster City, CA, U.S.A.). Finally, the targetted sequences were amplified in ABI 2720 PCR. PCR parameters were: 95°C for 4 min, 98°C for 20 s, 65°C for 30 s, 16 cycles, 72°C for 30 s, 72°C for 5 min, and 12°C hold. PCR reagents were KAPA Taq PCR Kits (KAPA Biosystems, Boston, Massachusetts, U.S.A.) and Nextflex primers were synthesized by Invitrogen (Thermo Fisher, Foster City, CA, U.S.A.). The quality of amplified library was determined by using an Agilent 2100 Bioanalyzer (Agilent Technologies, Santa Clara, CA, U.S.A.), and the library DNA concentration was again determined by Qubit dsDNA BR assay kit according to the manufacturer’s instructions. Libraries with good quality and DNA concentration >3 ng/μl were sequenced using an Illumina Hiseq X-ten sequencer (Illumina, San Diego, CA, U.S.A.).

### Bioinformatics analyses

#### Data QC

The adaptor sequences and raw reads with low quality reads were filtered using Trimmomatic software [21]. The adaptor sequences were GATCGGAAGAGCACACGTCT and AGATCGGAAGAGCGTCGTGTAGGGAAAGAGTGT, the criterion of low-quality sequence was the quality value of no more than Q20 or the accuracy of no more than 99%. The bases length should be longer than 40 bp after removing the base that does not meet the criteria. Finally fastqc software (http://www.bioinformatics.bbsrc.ac.uk/projects/fastqc/) was used to measure data quality to make sure that 95% of the remaining reads or clean reads with quality were more than Q30.

#### Variants calling

The clean reads were aligned to the reference human genome (February 2009, hg19, GRCh37, downloaded from UCSC) by BWA MEM software [22] to generate BAM files. In order to improve the accuracy, the samtools [23] and picard software (http://broadinstitute.github.io/picard/) were used to remove PCR repetitive sequence. The Genome Analysis Toolkit (GATK) [24] was used to detect the variants such as SNPs and InDels in BAM files. Finally, ANNOVAR software [25] was used to annotate the variants.

#### Functional effect of variants

Phenolyzer [26] was used to predict the association between the genotype and phenotype of HBV infection.

#### Association testing

Both single-variant and gene-based tests were performed. For single-variant based test, Fisher’s exact test was used to compare the different variants between HCC/control groups or subgroup, the false discovery rate (FDR) adjusted *P*-value <0.001 was considered significant. For gene-based tests, optimal unified approach for rare variant association test (SKAT-O) [27], a rare-variant association test used in small-sample case–control genome study, was used to assess excess-risk mutations in HCC/controls and subgroups. Default setting was used and significance were defined at FDR adjusted *P*-value <0.0001.

## Results

### Study subjects

A total of 53 HCC patients, 53 SH cases with previous HBV infection and 53 HCs were recruited, the clinical characteristics and statistics of study subjects were listed in Table 1.

**Table 1:** Demographics

	HCC	HC	SH
Gender, male/female	45/8	26/27	30/23
Age, M (Q)	56.0 (11.50)	34.0 (16.0)	55.0 (19.0)
Drinking, no/yes	40/13	46/7	43/10
Smoking, no/yes	41/12	46/7	41/12
HBV DNA, M (Q)	3200 (30842)	-	-
HbsAg, (+/−)	41/12	0/53	0/53
HbeAg, (+/−)	17/36	0/53	0/53
Anti-Hbe, (+/−)	7/11	-	-
ALT, M (Q)	38.0 (68.8)	-	-
AST, M (Q)	58.0 (72.5)	-	-
AFP, M (Q)	161.2 (1070.0)	-	-

Abbreviations: AFP, α-fetoprotein; ALT, Alanine aminotransferase; Anti-Hbe, antibody to HBeAg; AST, aspartate aminotransferase; HbeAg, HBV E antigen; HbsAg, HBV surface antigen; M, mean; Q, quartile.

### Targetted gene investigations

A total of 404 genes or coding region of ~500 kilo (K) bp, were sorted according to HGNC database, including the whole cytokine and receptor family genes, some innate immunity and adaptive immunity related genes which reported as pathogen sensors (Table 2). The cytokine and receptor family genes were: 43 interleukin (IL) family genes, 42 IL receptor genes, 21 interferon (IFN) genes, 5 IFN receptor genes, 45 chemokine ligand (CCL) genes, 24 chemokine receptor genes, 18 tumor necrosis factor genes, and 29 tumor necrosis factor receptor genes. The innate genes included 10 TLR genes, and 22 NLRs family genes. The adaptive immunity genes comprised 27 MHC-related genes, 18 T-cell inhibition and co-inhibition related genes, 28 T-cell activation and co-stimulation genes. Other immune response related genes were: 37 natural killer cell target genes, 54 G-protein-coupled receptor genes, and 16 other genes.

**Table 2:** Summary of genes investigated in the study

Group	Subgroup and gene numbers
Cytokines and receptors	ILs and receptors, *n*=85; IFNs and receptors, *n*=26; chemokines and receptors, *n*=69; tumor necrosis factor and receptors, *n*=47
Innate immunity	TLRs, *n*=10; NLRs, *n*=22
Adaptive immunity	MHCs, *n*=27; T-cell inhibitors, *n*=18; T-cell activators, *n*=28
Others	GPCRs, *n*=54; natural killer cell targets, *n*=37, others, *n*=16;

Abbreviation: GPCR, G-protein-coupled receptor.

### Targetted region sequencing performance

TRS yielded 10.84M clean reads (averaged read length was 143.65 bp) or 1577.94M clean bp for each sample, the average base quality was 40.26 and Q30 of call clean reads was 95.64%; the coverage rate of 499.968K region was 99.48%, duplication rate was 39.84%, and align rate was 99.23%; the capture rate was 50.53%. The target average depth was 919.55X, with target 10X rate was 99.31% and target 20X rate was 99.18%.

### Variants and genes related to HBV associated with HCC

**Figure 1:**
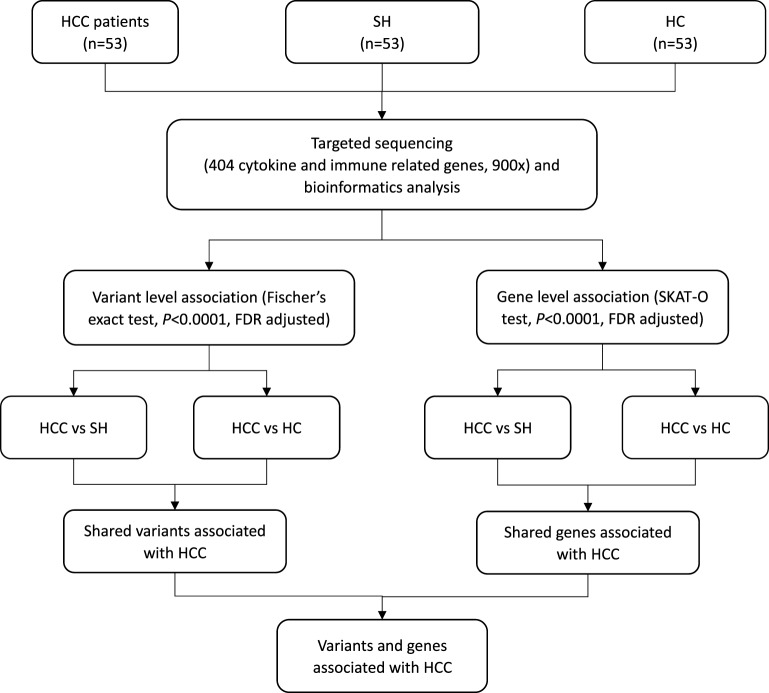
Flowchart of study design. CC, hepatocellular carcinoma with hapatitis B virus infection; FDR, false discovery rate; SH, self-healing cases with previous hepatitis B virus infection; HC, healthy controls; SKAT-O, optimal unified approach for rare variant association test.

As shown in flowchart (Figure 1), both HC and self-healing groups were used as controls to identify variants and genes associated with HCC. The results of variant and gene level association test for each subgroup were listed in Table 3.

**Table 3:** Association of variants and genes with HBV associated with HCC

Gene symbol	Associated SNVs	Variant level OR		Variant level *P*-value (FDR adjusted)		Numbers of SNVs	*SKAT-O* gene level *P*-value (FDR adjusted)	
		HCC compared with HC	HCC compared with SH	HCC compared with HC	HCC compared with SH		HCC compared with HC	HCC compared with SH
*HLA-B*	rs2596493	Inf	Inf	2.25E-18	3.59E-18	106	1.7232E-16	2.67597E-10
*HLA-B*	rs1131285	Inf	Inf	1.7E-13	1.13E-13	106	1.7232E-16	2.67597E-10
*HLA-B*	rs1065386	Inf	Inf	6.97E-09	6.97E-09	106	1.7232E-16	2.67597E-10
*HLA-B*	rs1050570	Inf	Inf	1.87E-08	1.83E-08	106	1.7232E-16	2.67597E-10
*HLA-B*	rs1050556	Inf	Inf	0.0000019	0.0000019	106	1.7232E-16	2.67597E-10
*HLA-B*	rs1131215	Inf	Inf	0.0000623	0.0000619	106	1.7232E-16	2.67597E-10
*HLA-C*	rs41556617	Inf	Inf	7.92E-12	6.79E-12	108	5.33706E-07	3.14762E-05
*HLA-C*	rs2308527	Inf	Inf	0.000000801	0.00000074	108	5.33706E-07	3.14762E-05
*HLA-DPA1*	rs1042174	16.63	28.69	0.000000899	3.15E-08	33	1.4521E-08	4.3979E-07
*HLA-DPA1*	rs1126543	Inf	Inf	2.9E-09	2.9E-09	33	1.4521E-08	4.3979E-07
*HLA-DPA1*	rs2308930	Inf	Inf	6.97E-09	6.97E-09	33	1.4521E-08	4.3979E-07
*HLA-DPA1*	rs2308929	Inf	Inf	6.97E-09	6.97E-09	33	1.4521E-08	4.3979E-07
*HLA-DPA1*	rs1126542	Inf	Inf	1.87E-08	1.83E-08	33	1.4521E-08	4.3979E-07
*HLA-DQA1*	rs9272896	Inf	Inf	0.0000116	0.0000113	81	3.33767E-07	1.30562E-05
*HLA-DQB1*	rs1049055	36.19	75.53	2.7E-11	6.3E-14	110	9.04603E-10	7.65036E-10
*HLA-DQB1*	rs1130430	19.53322682	11.3	0.00000465	0.0000835	110	9.04603E-10	7.65036E-10
*HLA-DQB1*	rs1049057	Inf	Inf	4.67E-08	4.18E-08	110	9.04603E-10	7.65036E-10
*HLA-DQB1*	rs1063345	Inf	Inf	0.00000465	0.0000045	110	9.04603E-10	7.65036E-10
*HLA-DQB1*	rs1140316	Inf	Inf	0.0000623	0.0000619	110	9.04603E-10	7.65036E-10
*HLA-DQB2*	rs9276572	Inf	Inf	2.05E-15	2.05E-15	21	5.04185E-06	0.000688766
*HLA-DRB1*	rs17211105	18.48	18.48	3.21E-08	3.02E-08	126	1.08223E-14	1.74002E-11
*HLA-DRB1*	rs9270299	12.56	16.04	0.00000974	0.00000051	126	1.08223E-14	1.74002E-11
*HLA-DRB1*	rs17883134	44.09	20.97	1.07E-10	1.83E-08	126	1.08223E-14	1.74002E-11
*HLA-DRB1*	rs9270303	20.97	20.97	1.95E-08	1.83E-08	126	1.08223E-14	1.74002E-11
*HLA-DRB1*	rs2308759	353.69	214.66	5.04E-20	3.59E-18	126	1.08223E-14	1.74002E-11
*HLA-DRB1*	rs17211091	18.04	96.28	0.000000363	1.61E-10	126	1.08223E-14	1.74002E-11
*HLA-DRB1*	rs113734598	48.46	15.62	0.00000159	0.0000619	126	1.08223E-14	1.74002E-11
*HLA-DRB1*	rs9269693	136.13	Inf	9.53E-13	3.74E-14	126	1.08223E-14	1.74002E-11
*HLA-DRB1*	rs1064697	Inf	Inf	0.000000124	0.000000107	126	1.08223E-14	1.74002E-11
*HLA-DRB1*	rs1071752	Inf	Inf	0.0000623	0.0000619	126	1.08223E-14	1.74002E-11
*HLA-DRB5*	rs17211043	Inf	Inf	0.000000124	0.000000107	83	4.77962E-10	2.52675E-06
*HLA-DRB5*	rs77853982	Inf	Inf	0.000000801	0.00000074	83	4.77962E-10	2.52675E-06
*HLA-DRB5*	rs701884	38.71	Inf	0.0000245	0.0000019	83	4.77962E-10	2.52675E-06
HLA-DRB5	rs1064587	35.87	Inf	0.000058	0.0000045	83	4.77962E-10	2.52675E-06
*HCAR2*	rs579877	Inf	Inf	0.0000277	0.000027	11	2.03163E-06	3.36441E-05
*HCAR2*	rs579876	Inf	Inf	0.0000277	0.000027	11	2.03163E-06	3.36441E-05
*NLRP11*	NM_145007: c.*131_*130delTG	9.684525939	14.70656392	2.71228E-05	6.95927E-07	22	6.83802E-07	2.97726E-09
*NLRP11*	rs368692979	17.64443693	32.53501457	8.81316E-08	8.54585E-10	22	6.83802E-07	2.97726E-09
*RAET1E*	NM_139165: exon5:c.623-2- >TT	16.6900097	20.59517629	1.0742E-07	9.68061E-09	21	0.000603407	3.07916E-05

Abbreviations: Inf, infinite; *HCAR2*, hydroxy-carboxylic acid receptor 2; OR, odds ratio; *NLRP11*, NLR protein 11; *RAET1E*, retinoic acid early transcript 1E; SH, self-healing cases with previous HBV infection.

A total of 39 significantly different variants and 11 genes were identified. Thirty four of the variants were from eight HLA genes, including *HLA-B, HLA-C, HLA-DPA1, HLA-DQA1, HLA-DQB1, HLA-DQB2, HLA-DRB1*, and *HLA-DRB5*. We also identified five non-HLA variants in retinoic acid early transcript 1E (*RAET1E*), NLR protein 11 (*NLRP11*), and *HCAR2*. NM_139165:exon5:c.623-2->TT was located in *RAET1E*. The variants identified were significantly enriched in HCC patients, and rare or no variants were found enriched in controls. rs368692979 and NM_145007:c.*131_*130delTG were located in *NLRP11*, and were two of the top ten most significant variants (*P*-values were 6.72E-14 and 7.08E-11 in HCC compared with HC and HCC compared with self-healing, respectively). Previous studies showed that *NLRP11* was related to inflammation [41]. These variants were also significantly enriched in HCC patients, and rare or no variants were found enriched in controls. rs579876 and rs579877 were located in 3′-UTR of *HCAR2*.

## Discussion

The present study found that 39 variants in 11 genes were strongly associated with HBV associated with HCC (HBV-HCC). Amongst them, 34 variants from eight genes in HLA region were previously described and our data were consistent with previous studies [30–35]. The novelties of our study are: five variants (rs579876, rs579877, rs368692979, NM_145007:c.*131_*130delTG, NM_139165:exon5:c.623-2->TT) from three genes (*REAT1E, NLRP11, HCAR2*) were found to be strongly associated with HBV-HCC. Our study provided fundamental data which needs further study to confirm the roles of these new variants and genes.

HBV infection is a serious public health problem [1]. Patients with HBV infection have different outcomes, such as self-healing, HBV carrier, chronic hepatitis, or HCC [4]. It has been found that the host genotype may be closely related to the different outcomes. Some HCC-related loci have been identified based on GWAS [10–14,28]. However, these studies are mainly based on the common variants, and most of the variants are located in the non-gene region. It is difficult to verify the gene function and explore the mechanism of disease. Given that HTS methods such as whole exome sequencing (WES) or whole genome sequencing (WGS) focus on not only common but also rare variants, HTS has been widely used in complex disease genetics studies and shows promising results. WES has been successfully applied in HCC genetics research [29].

Large sample size is necessary for HCC studies. However, WES-based GWAS is very expensive. TRS, which targets the specific gene families or pathway genes, provides relatively cost-effective solution. The present study investigated the cytokines and immune genes associated with HCC. We selected 404 genes that may be associated with HBV infection. These genes are mainly involved in antigen recognition, processing and presentation, immune regulatory cytokines and receptors, as well as those included in innate immune system, such as TLRs and NLRs. We have also studied some genes that are directly related to immune checkpoint.

We included SH cases because we speculated that they may carry less risk mutations or carry more protective mutations. Previous study demonstrated that the performance of gene level associated/burden test is reliable in WES/WGS study even in a small sample size in WES/WGS study [27]. The present study performed a comprehensive variants and gene level association analysis between both HCC compared with HC and HCC compared with SH. We found that 39 variants and 11 genes were strongly associated with HBV-HCC.

Not surprisingly, 34 variants from eight genes in HLA region were found strongly associated with HBV-HCC. Our data were consistent with previous studies [30–35]. We found five new variants from three genes that are also strongly related to HBV-HCC.

*RAET1E* is an MHC class I related gene from the RAET1 family, which functions as a ligand for the *NKG2D* receptor. *NKG2D* receptors are expressed on the surface of several types of immune cells and are involved in both innate and adaptive immune responses [36]. Previous studies showed that reduced *NKG2D* ligand expression in HCC correlates with early recurrence [37], and that the immunoreceptor *NKG2D* promotes tumor growth in a model of HCC [38]. Consistent with these studies, we found that a splicing variant in *RAET1E*, NM_139165:exon5:c.623-2->TT, might lead to malfunction of *RAET1E*, a ligand for the *NKG2D* receptor. As *NKG2D* receptor blockade is an attractive target for HCC therapy [39], further research will be necessary to study how *RAET1E* may be involved in HBV-associated HCC and develop candidate drugs.

*NLRP11* is a member of the *NLR* gene family, which is reported to be closely related to antiviral immunity [40]. Although little is known about whether *NLRP11* is involved in HBV infection, another member of the NLR family, *NLRP3*, is involved in danger signal inflammatory responses [41]. Furthermore, the four variants identified, including rs368692979 and NM_145007:c.*131_*130delTG in *NLRP11*, together with rs579876 and rs579877 in *HCAR2*, a gene closely related to immune activation [42], were all from the 3′-UTR region, which suggests that the expression of *NLRP11* and *HCAR2* might be under the regulation of miRNA.

## Conclusion

Our comprehensive investigation of cytokine- and immune-related gene mutations in HBV-HCC patients and controls showed that 39 different variants and 11 genes were significantly related to HBV-HCC. Our data implied that chronic hepatitis B patients who carry these variants need intensive monitoring.

## Abbreviations

BAM: Binary Alignment/Map
FDR: false discovery rate
GWAS: genome-wide association study
HbsAg: Hepatitis B surface antigen
HBeAg: Hepatitis B e antigen
HBcAb: Hepatitis B core antibody
HBeAb: Hepatitis B e antibody
HBV: hepatitis B virus
HBV-HCC: HBV associated hepatocellular carcinoma
HC: healthy control
HCAR2: hydroxy-carboxylic acid receptor 2
HCC: hepatocellular carcinoma
HGNC: HUGO Gene Nomenclature Committee
HLA: human leukocyte antigen
HTS: high-throughput sequencing
IFN: interferon
IL: interleukin
NLR: NOD-like receptor
NLRP11: NOD-like receptor protein 11
NOD: nucleotide-binding oligomerization domain
RAET: retinoic acid early transcript
RAET1E: retinoic acid early transcript 1E
SH: self-healing case
SNV: Single Nucleotide Variant
TLR: toll-like receptor
TRS: target region sequencing
QC: quality control
WES: whole exome sequencing
WGS: whole genome sequencing
